# Supplementary material for: Self-organization in brain tumors: How cell morphology and cell density influence glioma pattern formation
Source: PLoS Comput Biol. 2020 May 7;16(5):e1007611. doi: 10.1371/journal.pcbi.1007611 (PMC7244185; doi:10.1371/journal.pcbi.1007611)
Supplement: S1 Text — (PDF) [file pcbi.1007611.s001.pdf]

Supporting information: *Self-organization in brain tumors: how cell morphology and cell density influence glioma pattern formation*

Sara Jamous[1], Andrea Comba[2], Pedro R. Lowenstein[2,*], Sebastien Motsch[1,*]

1 Arizona State University, School of Mathematical & Statistical Sciences, Tempe, Arizona, USA
2 University of Michigan, Department of Neurosurgery Medical School, Ann Arbor, Michigan, USA

* pedrol@umich.edu, smotsch@asu.edu

Supporting information

Explicit expression of the model

We would like to provide an explicit expression of the dynamics by computing explicitly the gradient of the potential $\nabla_{\mathbf{x}_i} V_i$ and $\nabla_{\omega_i} V_i$. We first introduce the local coordinates:

$$x_{ij} = \langle \mathbf{x}_j - \mathbf{x}_i, \omega_i \rangle, \quad y_{ij} = \langle \mathbf{x}_j - \mathbf{x}_i, \omega_i^\perp \rangle \tag{1}$$

where $\omega_i^\perp$ is the orthogonal vector of ω_i. Simple computations show that:

$$\nabla_{\mathbf{x}_i} x_{ij} = -\omega_i \ , \ \nabla_{\mathbf{x}_i} y_{ij} = -\omega_i^\perp \ , \ \nabla_{\omega_i} x_{ij} = \mathbf{x}_j - \mathbf{x}_i \ , \ \nabla_{\omega_i} y_{ij} = -(\mathbf{x}_j - \mathbf{x}_i)^\perp.$$

We deduce that:

$$\begin{aligned}\nabla_{\mathbf{x}_i} V_i &= \sum_{j \neq i} \Phi'(r_{ij}^2) \nabla_{\mathbf{x}_i} r_{ij}^2 = 2 \sum_{j \neq i} \Phi'(r_{ij}^2) \left(\frac{x_{ij} \nabla_{\mathbf{x}_i} x_{ij}}{|a|^2} + \frac{y_{ij} \nabla_{\mathbf{x}_i} y_{ij}}{|b|^2} \right) \\ &= -2 \sum_{j \neq i} \Phi'(r_{ij}^2) \left(\frac{x_{ij}}{|a|^2} \, \omega_i + \frac{y_{ij}}{|b|^2} \, \omega_i^\perp \right), \end{aligned} \tag{2}$$

which provides an explicit expression to estimate $\mathbf{x}_i'$. To estimate ω_i', we compute similarly:

$$\begin{aligned}\nabla_{\omega_i} V_i &= 2 \sum_{j \neq i} \Phi'(r_{ij}^2) \left(\frac{x_{ij} \nabla_{\omega_i} x_{ij}}{|a|^2} + \frac{y_{ij} \nabla_{\omega_i} y_{ij}}{|b|^2} \right) \\ &= 2 \sum_{j \neq i} \Phi'(r_{ij}^2) \left(\frac{x_{ij} (\mathbf{x}_j - \mathbf{x}_i)}{|a|^2} - \frac{y_{ij} (\mathbf{x}_j - \mathbf{x}_i)^\perp}{|b|^2} \right). \end{aligned}$$

Using the explicit expression of the projection operator $P_{\omega_i^\perp} = \mathrm{Id} - \omega_i \otimes \omega_i$, we deduce:

$$P_{\omega_i^\perp}(\mathbf{x}_j - \mathbf{x}_i) = y_{ij} \cdot \omega_i^\perp \quad \text{and} \quad P_{\omega_i^\perp}\left((\mathbf{x}_j - \mathbf{x}_i)^\perp\right) = x_{ij} \cdot \omega_i^\perp. \tag{3}$$

Thus,

$$
\begin{aligned}
P_{\omega_i^\perp}(\nabla_{\omega_i} V_i) &= 2\sum_{j\neq i} \Phi'(r_{ij}^2)\left(\frac{x_{ij}y_{ij}\cdot\omega_i^\perp}{|a|^2} - \frac{y_{ij}x_{ij}\cdot\omega_i^\perp}{|b|^2}\right) \\
&= 2\sum_{j\neq i} \Phi'(r_{ij}^2)x_{ij}y_{ij}\left(\frac{1}{|a|^2} - \frac{1}{|b|^2}\right)\omega_i^\perp \\
&= -\frac{2e^2}{b^2}\sum_{j\neq i} \Phi'(r_{ij}^2)x_{ij}y_{ij}\,\omega_i^\perp. \qquad (4)
\end{aligned}
$$

In higher dimensions, i.e. $\mathbb{R}^3$, we cannot define anymore the local coordinate y_{ij} since $\omega_i^\perp$ is now an hyperplane. However, we can still define $x_{ij} = \langle \mathbf{x}_j - \mathbf{x}_i, \omega_i\rangle$ and we will use the second formulation for r_{ij}. Some elementary computations show that:

$$
\begin{aligned}
\nabla_{\mathbf{x}_i}\big[(\mathbf{x}_j - \mathbf{x}_i)\cdot\omega_i\big]^2 &= 2\big[(\mathbf{x}_j - \mathbf{x}_i)\cdot\omega_i\big](-\omega_i) = -2x_{ij}\omega_i, \\
\nabla_{\omega_i}\big[(\mathbf{x}_j - \mathbf{x}_i)\cdot\omega_i\big]^2 &= 2\big[(\mathbf{x}_j - \mathbf{x}_i)\cdot\omega_i\big](\mathbf{x}_j - \mathbf{x}_i) = 2x_{ij}(\mathbf{x}_j - \mathbf{x}_i).
\end{aligned}
$$

We deduce:

$$
\begin{aligned}
\nabla_{\mathbf{x}_i} V_i &= \sum_{j\neq i}\Phi'(r_{ij}^2)\nabla_{\mathbf{x}_i} r_{ij}^2 \\
&= \frac{1}{b^2}\sum_{j\neq i}\Phi'(r_{ij}^2)\left(\nabla_{\mathbf{x}_i}\|\mathbf{x}_j - \mathbf{x}_i\|^2 - e^2\nabla_{\mathbf{x}_i}\big[(\mathbf{x}_j - \mathbf{x}_i)\cdot\omega_i\big]^2\right) \\
&= \frac{1}{b^2}\sum_{j\neq i}\Phi'(r_{ij}^2)\left(2(\mathbf{x}_i - \mathbf{x}_j) + 2e^2 x_{ij}\omega_i\right).
\end{aligned}
$$

Similarly, we have:

$$
\begin{aligned}
\nabla_{\omega_i} V_i &= \sum_{j\neq i}\Phi'(r_{ij}^2)\nabla_{\omega_i} r_{ij}^2 \\
&= \frac{1}{b^2}\sum_{j\neq i}\Phi'(r_{ij}^2)\left(\nabla_{\omega_i}\|\mathbf{x}_j - \mathbf{x}_i\|^2 - e^2\nabla_{\omega_i}\big[(\mathbf{x}_j - \mathbf{x}_i)\cdot\omega_i\big]^2\right) \\
&= \frac{2e^2}{b^2}\sum_{j\neq i}\Phi'(r_{ij}^2)x_{ij}(\mathbf{x}_i - \mathbf{x}_j).
\end{aligned}
$$

Using that the projector operator $P_{\omega_i^\perp} = \mathrm{Id} - \omega_i \otimes \omega_i$, we deduce:

$$
P_{\omega_i^\perp}(\mathbf{x}_j - \mathbf{x}_i) = (\mathbf{x}_j - \mathbf{x}_i) - [(\mathbf{x}_j - \mathbf{x}_i)\cdot\omega_i]\cdot\omega_i = (\mathbf{x}_j - \mathbf{x}_i) - x_{ij}\cdot\omega_i
$$

Thus,

$$
P_{\omega_i^\perp}(\nabla_{\omega_i} V_i) = -\frac{2e^2}{b^2}\sum_{j\neq i}\Phi'(r_{ij}^2)x_{ij}\left((\mathbf{x}_j - \mathbf{x}_i) - x_{ij}\cdot\omega_i\right).
$$

Notice that in any dimension, if the cell i has a circular shape (i.e. $a = b$ and the eccentricity becomes $e = 0$), then $\dot{\omega}_i = -\beta P_{\omega_i^\perp}(\nabla_{\omega_i} V_i) = 0$. Thus, the dynamics will have no effect on the orientation of the cell.

Nematic average velocity

Flocking pattern is primary described using average velocity from which we deduce the polarization of the flock. Denoting $(\omega_i)_{i=1..N}$ the velocity of all the cells, the average velocity $\langle\omega_i\rangle$ and polarization ψ are given by:

$$\langle\omega_i\rangle_i = \frac{1}{N}\sum_{i=1}^{N}\omega_i \quad \text{and} \quad \psi = |\langle\omega_i\rangle_i|.$$

However, for streaming formation, it is more difficult to define the average *nematic velocity* velocity or the average *nematic direction*. With this aim, we need to introduce an optimization problem. For instance, the direction of the average velocity can be seen as the maximization of:

$$\max_{\Omega\in\mathbb{S}^{d-1}} \frac{1}{N}\sum_{i=1}^{N}\langle\omega_i,\Omega\rangle.$$

By Cauchy-Schwarz inequality, the maximizer Ω_* must be in the direction of the average $\langle\omega_i\rangle_i$. We would like to define similarly the average nematic direction as the direction Ω_{nem} that maximizes the function:

$$J(\Omega) = \frac{1}{N}\sum_{i=1}^{N}\langle\omega_i,\Omega\rangle^2. \tag{5}$$

Notice in particular that $J(\Omega) = J(-\Omega)$.

Nematic average in $\mathbb{R}^2$. Denote θ_i the angle between the velocity ω_i of the cell i and the horizontal axis. Also, define the angle $\bar{\theta}$ of the maximizer Ω_{nem} with respect to the x-axis. Notice that:

$$\max_{\Omega\,\in\,\mathbb{S}^1} J(\Omega) \quad = \quad \max_{\Omega\,\in\,\mathbb{S}^1} \frac{1}{N}\sum_{i=1}^{N}\langle\omega_i,\,\Omega\rangle^2 = \max_{\theta}\frac{1}{N}\sum_{i=1}^{N}\cos^2(\theta_i-\theta).$$

To maximize J, we must solve $\frac{\partial J(\bar{\theta})}{\partial\theta} = 0$. We find:

$$\begin{aligned}
\frac{\partial J(\bar{\theta})}{\partial\theta} &= \frac{1}{N}\sum_{i=1}^{N} 2\cos\left(\theta_i-\bar{\theta}\right)\sin\left(\theta_i-\bar{\theta}\right) = \frac{1}{N}\sum_{i=1}^{N}\sin\left(2\left(\theta_i-\bar{\theta}\right)\right) \\
&= \frac{1}{N}\sum_{i=1}^{N}\left[\,\sin(2\theta_i)\cos\left(2\bar{\theta}\right) - \cos(2\theta_i)\sin\left(2\bar{\theta}\right)\,\right] \\
&= \langle\sin(2\theta_i)\rangle_i\cos\left(2\bar{\theta}\right) - \langle\cos(2\theta_i)\rangle_i\sin\left(2\bar{\theta}\right).
\end{aligned}$$

Therefore, $\frac{\partial J(\bar{\theta})}{\partial\theta} = 0$ leads to:

$$\frac{\langle\sin(2\theta_i)\rangle_i}{\langle\cos(2\theta_i)\rangle_i} = \frac{\sin\left(2\bar{\theta}\right)}{\cos\left(2\bar{\theta}\right)}.$$

This motivates the following definition.

Definition 1 *The nematic average direction Ω_{nem} is defined as:*

$$\Omega_{nem} = (\cos\bar{\theta},\,\sin\bar{\theta}) \qquad \textit{with} \quad \bar{\theta} = \frac{1}{2}\arctan\left(\frac{\langle\sin(2\theta_i)\rangle_i}{\langle\cos(2\theta_i)\rangle_i}\right). \tag{6}$$

Remark 2 *If we denote the nematic average:*

$$\mathbf{u}_{nem} = \big(\langle\cos(2\theta_i)\rangle_i,\ \langle\sin(2\theta_i)\rangle_i\big)^T \tag{7}$$

the nematic polarization is given by $\gamma = |\mathbf{u}_{nem}|$*. This formula is similar to the expression of the usual polarization* ψ *giving as the norm of the average velocity* $|\langle\omega_i\rangle_i|$*.*

Nematic polarization in higher dimension. Note that the previous section is only applicable in $\mathbb{R}^2$ since we use polar coordinates. To find the maximizer Ω_* of J (5) in higher dimension, we introduce the Lagrangian:

$$\mathcal{L}(\Omega,\lambda) = J(\Omega) - \lambda g(\Omega)$$

with $g(\Omega) = |\Omega|^2 - 1$. The maximizer Ω_{nem} must be a critical point to $\mathcal{L}$ which leads to the existence of a Lagrange multiplier λ_* satisfying:

$$\begin{aligned} \nabla_\Omega J(\Omega_*) = \lambda_* \nabla_\Omega g(\Omega_*) \quad &\Rightarrow \quad \frac{2}{N}\sum_{i=1}^N \langle\omega_i,\Omega_*\rangle\omega_i = 2\lambda_*\Omega_* \\ &\Rightarrow \quad \frac{1}{N}\sum_{i=1}^N [\omega_i\otimes\omega_i]\Omega_* = \lambda_*\Omega_*. \end{aligned}$$

Denoting the matrix $A = \frac{1}{N}\sum_{i=1}^N[\omega_i\otimes\omega_i]$, we deduce that λ_* and Ω_* are eigenvalue/eigenvector of A. Therefore, J is maximized at $\Omega_\star$ eigenvector associated to the largest eigenvalue of the matrix A.

Remark 3 *A is a positive semi-definite matrix, so the quantity to be maximized J is recognized as a Rayleigh quotient.*

Remark 4 *In dimension larger than 2, the nematic average vector* $\mathbf{u}_{nem}$ *(7) is no longer defined. Thus, we cannot define nematic polarization as* $\gamma = |\mathbf{u}_{nem}|$*. Instead, we can use as statistics the value of J at the maximizer* Ω_{nem}*. Similarly, we find that* $J(\Omega_{nem})$ *is close to 1 when the vectors form a stream and zero if there are distributed uniformly.*

Moreover, in $\mathbb{R}^2$*, we observe that the nematic polarization γ and* $J(\Omega_{nem})$ *are related to each other since:*

$$\begin{aligned} J(\Omega_*) &= \frac{1}{N}\sum_{i=1}^N\langle\omega_i,\,\Omega\rangle^2 = \frac{1}{N}\sum_{i=1}^N\cos^2(\theta_i-\bar\theta) = \frac{1}{2N}\sum_{i=1}^N\big(1+\cos\big(2(\theta_i-\bar\theta)\big)\big) \\ &= \frac{1}{2}+\frac{1}{2}\langle\cos(2(\theta_i-\bar\theta))\rangle_i = \frac{1}{2}+\frac{1}{2}\big[\langle\cos(2\theta_i)\rangle_i\cos(2\bar\theta)+\langle\sin(2\theta_i)\rangle_i\sin(2\bar\theta)\big] \\ &= \frac{1}{2}+\frac{1}{2}|\mathbf{u}_{num}| \end{aligned}$$

since $\mathbf{u}_{num}$ *and the vector* $(\cos 2\bar\theta,\,\sin 2\bar\theta)^T$ *are parallel. Thus:*

$$J(\Omega_*) = \frac{1}{2}+\frac{1}{2}\gamma \quad \Rightarrow \quad \gamma = 2J(\Omega_*)-1.$$

Therefore, using γ or $J(\Omega_*)$ *provide similar information about the nematic alignment of the cells. However, the advantage of* $J(\Omega_*)$ *is to generalize in any dimensions.*
