# Supplementary material for: Self-organization in brain tumors: How cell morphology and cell density influence glioma pattern formation
Source: PLoS Comput Biol. 2020 May 7;16(5):e1007611. doi: 10.1371/journal.pcbi.1007611 (PMC7244185; doi:10.1371/journal.pcbi.1007611)
Supplement: Attachment — Submitted filename: letter_reviewers.pdf [file pcbi.1007611.s004.pdf]

Arizona State University

Physical Sciences Building A-Wing Rm. 836
School of Mathematical & Statistical Sciences
Tempe, AZ 85287-1804, USA

Sebastien Motsch
Associate Professor
School of Math. & Stat. Sciences

email : smotsch@asu.edu

March 1st, 2020

PLOS Computational biology

Dear Drs. Maini and Mac Gabhann,

We thank you very much for your kind letter. We are delighted that contingent on our answering the reviewers, you will be likely to accept our manuscript for publication in PLOS Computational Biology.

We are hereby submitting all necessary materials according to reviewers', editors', and editorial comments. We trust this will allow you to proceed with the processing of our manuscript.

Yours sincerely,

Sebastien Motsch

Revision: Self-organization in brain tumors: how cell morphology and cell density influence glioma pattern formation

Sara Jamous, Andrea Comba, Pedro Lowenstein, Sebastien Motsch

1 Reviewer # 1

- *"The authors propose a novel agent-based model (ABM) that simulates patterns of self-organization of elliptic/ellipsoid-shaped glioma cells in 2 & 3D. This is an exciting contribution and the authors are correct that studying the complex dynamics of such emergent flocks and (onco)streams – cells moving in the same/opposite directions - may potentially hold great promise for assisting in the development of innovative cytoreductive and cytotoxic therapeutic strategies, much needed for these extremely difficult to treat brain neoplasms."*

  We thank the reviewer for his positive comments on our manuscript, and his encouraging view that these efforts on understanding glioma dynamics may potentially lead to the development of new therapies for this very aggressive cancer.

- *"Like any other model, this one has its limitations as e.g. the absence of biochemical factors reduces its levers to biomechanics, which is perfectly ok as long as one keeps this in mind when evaluating the results. For instance, the authors correctly emphasize that it is contrary to most concepts that an increase in cell density would lead to a reduction in flocks, and present this as an unexpected, noteworthy finding. The difficulty I am having with this is that real cancer cells are characterized by what is known as a loss of 'contact inhibition' ... in other words, they exhibit the opposite of such 'cell-cell avoidance'. It is precisely this feature that allows cells to form 2D-cell layer clusters in dishes and multicellular tumor spheroids in gels. In this context, the authors perhaps may find this paper an interesting read:*

  https://onlinelibrary.wiley.com/doi/abs/10.1046/j.1365-2184.2001.00202.x"

  We agree with this reviewer that all models have limitations, and that our model's effects are reduced to the consequences of cell shape and density on intratumoral dynamics. However, our model could still generate flocking/stream pattern at large density if one would reduce the self-propulsion of the cells (i.e. reducing the parameter c). As we illustrated in figure 9, the self-propulsion is acting like a perturbation to the 'energy' decay dynamics. Thus, reducing the speed would reduce this perturbation and hence allows the dynamics to converge to energy minimizers (i.e flocks or streams). In short, adding 'speed inhibition' would allow "flock" formation at high density. We added a discussion in the conclusion.

  We thank the reviewer for pointing out the paper in Cell Proliferation. This paper is now cited in the references and discussed in the main text of our manuscript. Specifically, using an in vitro spheroid system, this paper proposes pattern formation in tumor systems related to cell proliferation and invasion. Our manuscript mainly presents the results of a new model to help explain the underlying mechanisms that lead to the formation of flocks and streams in vivo. We have previously shown that both flocks and streams exist in vivo (Lowenstein, Motsch, Abstract Am Soc Cell Biol 2015; and manuscript in preparation).

- *"For the sake of the argument, once the import of cell-cell avoidance is reversed and perhaps (in form of PDEs in a discrete-continuum extension of this work) one would introduce biochemical factors secreted by the agents/cells (autocrine, with paracrine impact) in response to microenvironmental triggers, cells at higher N [a non-explicit representation of either a replication capability of the agents (say depending on space-lattice availability in this biomechanical setting) or a dynamic Tumor growth term, N_t which are both missing here, say Gompertz or Logistic] would likely display more same-direction flock and less opposite-direction streaming behavior, particularly as N increases, which is realistic in a spatially confined area (3D tissue, skull); similarly, the impact of (different cell size/shape) 'packing' – an exciting extension of this work, correctly mentioned on pg. 13 - will likely be less pronounced in this cell-cell avoidance setting. Rather, one would think that securing cell outflow from a rapidly proliferating tumor surface is critical to manage the onset of central apoptosis and necrosis (see clinical MRIs with circular contrast enhancement) which in turn is to some degree a function of dwindling vascular support across the surface. In this 3/4D scenario, what would be interesting to study – with a model such as this one - is if directionality, steering angle & velocity/speed change as a function of the distance to the main tumor – with the hypothesis that the closer the individual cell is to the main tumor, in an area of metabolic constraint and reduced tissue carrying capacity, the more likely it is to follow others departing in the same direction (away from the tumor), with a straight angle at higher speed – while at some distance, in a nutrient-rich environment with less mechanical confinement through the growing main mass, sparsely seeded invasive cells could explore multi-directionality and angles with higher degrees of freedom to forage (– analogous to complex systems in social insects like ants). This would likely result in fractal like invasive patterns that we have seen in gels and that (probably reinforced by fractal-like neovascular architecture) have been noted for real tumor boundaries in the past (see e.g. works by Cross S.S.)."*

  We agree that it will be interesting to investigate how the tumor spreads if one includes cell division at a particular location (e.g. center of the tumor). It is especially challenging to guess if the spreading would be faster or slower at center or periphery of the tumor. One could argue that the spreading would be slower at the periphery since the density is too low and thus no flocking pattern emerge. But similarly flocks do not occur at large density. Thus we need future experimentations. Also, one would need to take into account the complex changes in the mechanical characteristics of the tissue being invaded, especially as important changes in stiffness occur at the border, and cells have a preference for increased stiffness. We added a discussion concerning this relevant question in the conclusion.

  Since these suggestions would imply major changes to our model and manuscript, we would prefer to implement these in future iterations of our model.

- *"In summary, to put the claim of this work i.e. having clinical potential into perspective, I would like to encourage the authors to discuss key limitations of their current work, both in setup and results, in more detail."*

  In reviewing this manuscript according to these comments by reviewer 1 we have further expanded the discussion of key limitations of our current work and model in more detail, as requested.

- *"Finally, as a minor item, in Figure 1B, it appears that the blue labels being used to denote 'cells' – but this is an H&E staining and convention is that it depicts cell 'nuclei' in blue, cell bodies in pink. Perhaps I misunderstood, but worth clarifying."*

In answer to this comment, cells in Figure 1B are indeed labeled with H&E and display darker blue colors in the nuclei, and pink cell bodies. However the circles and ellipses had been drawn to outline the nuclei. Nuclei of eccentric cells are themselves eccentric, and nuclei of circular cells are themselves circular. In the revised manuscript we have amended the cell outlines to include both the nuclei and the cytoplasm as suggested.

2 Reviewer # 2

- *"The authors study an important question in tumor biology in general and glioma in particular. They investigate the effects of cell shape and density on organization and direction of movement. The model is elegant and the results are interesting. The presentation and Figures are clear."*

  We thank the reviewer for pointing out that our manuscript addresses and important question in tumor biology and in glioma biology, that our model is elegant and the results interesting, and that the Figures are clear.

- *"I have minor suggestions/questions:*
  1. Did the author observe the existence of streams in experimental data? Figure 1 is static; though some cells are elongated, the direction of movement can not be determined."

  Yes, we did observe the existence of streams and flocks in in vivo glial tumors imaged continuously over 2-3 days. These data were presented earlier in Lowenstein, Motsch, ASCB, 2015 (*Streams, swirls, and neurospheres: in vivo self-organization of brain tumors revealed by mathematical and biological modeling*), and Comba et al., manuscript in preparation.

- *"2. The dynamic behavior of circular cells is not surprising as it is predetermined by the equations; see page 4, lines 106-109. I suggest mentioning this point in the discussion."*

  We thank the reviewer for this comment, which is now mentioned in the discussion.

- *"3. I suggest combining Figures 10-12 into a single Figure."*

  In reply to this comment, we have now combined Figures 10-12 into one single figure.
